# *Trichoderma* Biocontrol: Signal Transduction Pathways Involved in Host Sensing and Mycoparasitism

**DOI:** 10.4137/grsb.s397

**Published:** 2007-11-08

**Authors:** Susanne Zeilinger, Markus Omann

**Affiliations:** Vienna University of Technology, Institute for Chemical Engineering, Research Area of Gene Technology and Applied Biochemistry, Working Group Molecular Biochemistry of Fungi, Getreidemarkt 9, A-1060 Vienna, Austria

**Keywords:** Trichoderma, biocontrol, mycoparasitism, signal transduction

## Abstract

Fungi of the genus *Trichoderma* are used as biocontrol agents against several plant pathogenic fungi like *Rhizoctonia* spp., *Pythium* spp., *Botrytis cinerea* and *Fusarium* spp. which cause both soil-borne and leaf- or flower-borne diseases of agricultural plants. Plant disease control by *Trichoderma* is based on complex interactions between *Trichoderma*, the plant pathogen and the plant. Until now, two main components of biocontrol have been identified: direct activity of *Trichoderma* against the plant pathogen by mycoparasitism and induced systemic resistance in plants. As the mycoparasitic interaction is host-specific and not merely a contact response, it is likely that signals from the host fungus are recognised by *Trichoderma* and provoke transcription of mycoparasitism-related genes.

In the last few years examination of signalling pathways underlying *Trichoderma* biocontrol started and it was shown that heterotrimeric G-proteins and mitogen-activated protein (MAP) kinases affected biocontrol-relevant processes such as the production of hydrolytic enzymes and antifungal metabolites and the formation of infection structures. MAPK signalling was also found to be involved in induction of plant systemic resistance in *Trichoderma virens* and in the hyperosmotic stress response in *Trichoderma harzianum*. Analyses of the function of components of the cAMP pathway during *Trichoderma* biocontrol revealed that mycoparasitism-associated coiling and chitinase production as well as secondary metabolism are affected by the internal cAMP level; in addition, a cross talk between regulation of light responses and the cAMP signalling pathway was found in *Trichoderma atroviride*.

## Introduction

Fungal species of the genus *Trichoderma* occur worldwide and can be isolated from soil, decaying wood and other forms of plant organic material. Mycoparasitic *Trichoderma* species are used commercially as biological control agents against plant-pathogenic fungi such as *Rhizoctonia solani, Botrytis cinerea, Sclerotium rolfsii, Sclerotinia sclerotiorum*, *Pythium* spp., and *Fusarium* spp. in, among others, the United States, India, Israel, New Zealand, and Sweden as a promising alternative to chemical pesticides (Barak and Chet, 1986; Chet, 1987; Harman and Björkman, 1987; Howell, 2003).

What currently is defined as biocontrol is a combination of different mechanisms working synergistically to achieve disease control (Howell, 2003). *Trichoderma* mycoparasitism combines processes such as nutrient competition (Chet, 1987), the secretion of antifungal metabolites (e.g. Dennis and Webster, 1971; Claydon et al. 1987; Schirmböck et al. 1994; Lorito et al. 1996) and formation of morphological changes such as coiling around the host and development of appressorium-like structures (Elad et al. 1983; Lu et al. 2004). As mycoparasitism by *Trichoderma* results in penetration of the cell wall of the host fungus and utilization of its cellular contents, hydrolytic enzymes such as chitinases, glucanases, and proteases, which are at least partially induced before direct contact with the host, play major roles in biocontrol (Hjeljord and Tronsmo, 1998). Chitinase gene expression is induced in liquid culture by e.g. fungal cell walls, colloidal chitin, or the chitin monomer N-acetylglucosamine (Kubicek et al. 2001). In *Trichoderma atroviride* the N-acetylglucosaminidase-encoding gene *nag1* has a major impact on the induction by chitin of other chitinases (Brunner et al. 2003). In mycoparasitic interactions between *Trichoderma* and *R. solani*, a diffusible factor released from the host is responsible for induction of *ech42* (endochitinase 42-encoding) gene transcription before physical contact (Cortes et al. 1998; Zeilinger et al. 1999). Upon direct contact, lectins in the host’s cell wall can induce coiling of the mycoparasite around the host hyphae (Barak and Chet, 1986; Inbar and Chet, 1994). The current model suggests that both enzyme production and infection structure formation are induced responses triggered by molecules released from the host fungus (e.g. degradation products from its cell wall) or located on its surface (e.g. lectins) (Inbar and Chet, 1992; Zeilinger et al. 1999).

The expression of genes associated with bio-control appears to be regulated by intracellular signal transduction pathways, which are activated by the binding of host-derived ligands to as yet unidentified receptors. The elucidation of these pathways has recently begun and has confirmed the involvement of highly conserved signalling components.

## The Role of G Protein Signalling in *Trichoderma* Biocontrol

Heterotrimeric G protein signalling is basically comprised of three parts: a G protein-coupled receptor (GPCR), a heterotrimeric G protein (α, β, γ subunits), and an effector (Neer, 1995). More than 20 years ago, the first GPCR-encoding gene was cloned and now more than 1000 have been described and characterized, mainly of vertebrate origin (Kolakowski, 1994). The overall amino acid sequence similarities between GPCRs are low, but these receptor proteins all have in common seven transmembrane domains and have the N-terminus outside and the C-terminus inside the cytoplasm. Ligand binding to the receptor results in a conformational change leading to release of the G protein and exchange of GDP for GTP on the Gα subunit. GTP-bound α dissociates from its βγ partner, allowing both signalling units to regulate the activities of downstream effectors (Kaziro et al. 1991; Birnbaumer et al. 1992; Neer, 1995; Gutkind, 1998).

Due to only minimal amino acid similarities between GPCRs, intensive research depended on the release of genome sequences. Numerous fungal genomes are available nowadays and comparative genomics pointed out that receptors can be classified into nine groups (Lafon et al. 2006): classes I and II comprise pheromone receptors with similarity to the yeast Ste2p and Ste3p receptors; classes III and V contain putative carbon source receptors and cAMP-sensors; class IV comprises *Schizosaccharomyces pombe* Stm1p-like nitrogen sensors; class VI, which is characteristic of filamentous fungi, comprises receptors with an RGS domain downstream of their transmembrane regions; classes VII and VIII have been identified only recently and share similarities with some vertebrate receptors; class IX comprises fungal opsins, similar to the bacterial retinal-binding rhodopsin, with *Neurospora crassa* NOP-1 and ORP-1 as prominent members (Borkovich et al. 2004). Preliminary investigations of the *Trichoderma reesei* (http://genome.jgi-psf.org/Trire2/Trire2.home.html) and *T. atroviride* (unpublished; release in progress) genomes revealed at least 16 putative proteins with 7-transmembrane domains, well distributed over all nine receptor classes (Brunner and Zeilinger, unpublished).

Highly conserved heterotrimeric G-proteins act as signal transducers that couple cell surface receptors to cytoplasmic effector proteins. In fungi, G-proteins are essential during sexual and pathogenic development and during secondary metabolism. They are part of the pheromone signalling cascade and also affect a number of developmental and morphogenetic processes which determine the virulence of fungi and plant fungal pathogens (e.g. Bölker, 1998).

G-protein α subunits can be classified into three major subgroups (Bölker, 1998). Members of subgroup I are homologues of the mammalian Gα_i_ subunits that inhibit adenylate cyclase (Turner and Borkovich,1993). Only a few members of subgroup II of fungal Gα proteins, which are characterized by the lack of the consensus site for pertussis toxin-dependent ribosylation, have so far been associated with a biological function or a distinct phenotype. Members of the fungal subgroup III, which are homologues of the mammalian Gα_s_ family, have been implicated to positively influence the internal cAMP level (Bölker, 1998).

Rocha-Ramirez et al. (2002) analyzed the *T. atroviride* subgroup I Gα subunit Tga1 by *tga1* over-expression and *tga1* gene silencing and showed that it is involved in both coiling and conidiation. *tga1* gene deletion confirmed these findings but revealed that some of the observed effects were more distinct in the deletion mutant (Reithner et al. 2005). Further characterisation of the Δ*tga1* mutant showed that this G-protein α subunit affects processes like vegetative growth, production of antifungal metabolites, and chitinase formation (Reithner et al. 2005), which are at least partially involved in *Trichoderma* biocontrol. In liquid culture the Δ*tga1* mutant produced strongly decreased chitinase activities and showed a reduced transcription of the *nag1* (N-acetyl-glucosaminidase-encoding) and *ech42* (endochitinase 42-encoding) genes (Reithner et al. 2005). In antagonistic assays, the Δ*tga1* mutant was unable to overgrow and lyse host fungi such as *R. solani, B. cinerea*, and *S. sclerotiorum*, although infection structure formation was unaffected; nevertheless, it displayed an enhanced growth inhibition of the host fungi by over-producing and secreting low molecular weight metabolites. To get insights into the nature of these over-produced substances which caused the increase in host growth inhibition, the production of some main secondary metabolites produced by *T. atroviride* was analyzed. Interestingly, the production of 6-pentyl-α-pyrone and of metabolites with sesquiterpene structure was reduced in the Δ*tga1* mutant (Reithner et al. 2005), while it produced elevated amounts of peptaibols belonging to the trichorzianine family (Stoppacher, Zeilinger, Schuhmacher, unpublished), suggesting opposite roles of Tga1 in regulating the biosynthesis of different antifungal substances in *T. atroviride*.

In contrast to the role of Tga1 in influencing growth and conidiation in *T. atroviride,* its homologue TgaA did not affect these properties in *Trichoderma virens.* Δ*tgaA* mutants grew normally and sporulated like the wild type, but had a reduced ability to colonise *S. rolfsii* sclerotia, whereas they were fully pathogenic against *R. solani* (Mukherjee et al. 2004). No such host specificity could be observed in the *T. atroviride* Δ*tga1* mutant.

Mutants of *T. virens* lacking the TgaB protein (belonging to subgroup II Gα subunits) did not show major phenotypic defects: they grew and sporulated like the wild type and biocontrol against *R. solani* and sclerotia of *S. sclerotiorum* was unaffected (Mukherjee et al. 2004).

Characterization of *T. atroviride* Tga3 revealed involvement of this subgroup III Gα subunit in regulating vegetative growth, conidiation, and conidial germination (Zeilinger et al. 2005). Δ*tga3* mutants had reduced intracellular cAMP levels and were avirulent in direct confrontation assays against *R. solani* or *B. cinerea.* In addition, mycoparasitism-related infection structures were not formed, strongly suggesting a loss of host recognition. Δ*tga3* mutants produced reduced extracellular chitinase activity even though the chitinase-encoding genes *ech42* and *nag1* were transcribed at a significantly higher rate than they were in the wild type. The observed accumulation of chitinolytic enzymes inside the cell and their retention in the cell wall may be due to an influence of Tga3 on chitinase secretion. Addition of exogenous cAMP did not suppress the altered phenotype or restored mycoparasitic overgrowth, although it did restore the ability to produce infection structures (Zeilinger et al. 2005). *In planta* biocontrol assays confirmed that Δ*tga3* mutants were unable to protect bean plants against infection with *R. solani* (Lorito and Zeilinger, unpublished).

## Mitogen-Activated Protein Kinases in *Trichoderma* Biocontrol

A variety of signals are transduced by mitogen-activated protein kinase (MAPK) cascades through sequential activation of serine/threonine protein kinases by phosphorylation resulting in the control of gene expression required by a plurality of biological processes in eukaryotes (Banuett, 1998; Schaeffer and Weber, 1999). MAPK cascades are evolutionarily conserved in all eukaryotes: they are typically organised in a three kinase architecture consisting of a MAPK, a MAPK activator (MEK, MKK or MAPK kinase) and a MEK activator (MEK kinase = MEKK or MAPK kinase kinase). In yeasts there are five MAPK genes transmitting signals for mating, filamentous growth, cell integrity, response to osmotic stress, and ascospore formation (Gustin et al. 1998). In fungi, genes encoding MAPK homologues are essential for developmental processes such as sporulation, mating, hyphal growth, and pathogenicity (Xu, 2000); nevertheless, examples of complete MAP kinase modules examined in filamentous fungi are rare.

The best studied MAPKs in *Trichoderma* belong to the family of yeast and fungal extracellular-related kinases (YERK1), a class also comprising MAPKs such as Pmk1 from *Magnaporthe grisea*, Fmk1 from *Fusarium oxysporum*, Bmp1 from *B. cinerea*, or Ubc3/Kpp2 from *Ustilago maydis*.

The *T. virens* MAPK homolog belonging to the YERK1 class was described by two different groups: although *tmkA* and *tvk1* encode the same protein in different strains of *T. virens*, contradictory results concerning the role of this MAP kinase in the production of mycoparasitism-related enzymes have been reported (Mendoza-Mendoza et al. 2003; Mukherjee et al. 2003). Enzyme activities of chitinases and proteases were described to be elevated in Δ*tvk1* mutants, but Δ*tmkA* strains showed a delay and reduction in clearing a chitin-containing medium. Moreover, Mukherjee et al. (2003) postulated that Δ*tmkA* mutants lost their biocontrol potential in a host-specific manner as they exhibited mycoparasitic coiling and lysis of *R. solani* hyphae similar to the wild type *T. virens* IMI304061, whereas they had reduced antagonistic properties in confrontation assays against *S. rolfsii* and they failed to parasitize the sclerotia of this pathogen. On the other hand, Δ*tvk1* mutants of *T. virens* Gv29-8 were reported to show a clear increase in the expression level of mycoparasitism-related genes during direct confrontation with *R. solani* and they were described to be more effective in disease control than the wild type or the chemical fungicide Apron (Mendoza-Mendoza et al. 2003). Further studies on the role of Tvk1 revealed that this MAPK regulates conidiation, hydrophobicity and the expression of genes coding for cell wall proteins during development of *T. virens* (Mendoza-Mendoza et al. 2007).

Recently, the corresponding MAPK from *T. atroviride* (Tmk1) was also characterized and showed 98% identity with *T. virens* TmkA/Tvk1 (Reithner et al. 2007). Δ*tmk1* mutants exhibited decreased radial growth and showed light-independent conidiation. Results from direct plate confrontation assays against *R. solani* and *B. cinerea* as hosts suggested that *T. atroviride* Tmk1 – similar to *T. virens* TmkA – affected the host specificity as Δ*tmk1* mutants still could parasitize *R. solani* (although less effectively than the parental strain) whereas they were unable to attack *B. cinerea*. Microscopic analysis of hyphae of the *T. atroviride* Δ*tmk1* mutants during interaction with *R. solani* revealed that they were still able to attach and coil around the host. Moreover, deletion of *tmk1* caused an extensive, host-independent coiling around the own hyphae and attachment to plain Nylon fibers. In Δ*tmk1* mutants, *nag1* and *ech42* transcript levels as well as extracellular chitinase activities were significantly elevated under chitinase-inducing conditions and the mutants also showed a de-regulation of the production of antifungal metabolites (e.g. increased production of 6-pentyl-α-pyrone and peptaibols). Consistent with the finding that mycoparasitism-related processes, such as infection structure formation (coiling) and chitinase and antifungal metabolite production, were enhanced upon *tmk1* gene deletion, Δ*tmk1* mutants showed an improved ability to protect bean plants against *R. solani* infection (Reithner et al. 2007).

It has recently been recognized that the basis for plant protection by *Trichoderma* is not only the direct mycoparasitic interaction with the pathogen but also a systemic defence response induced in the plant by colonization of its roots by *Trichoderma* (Harman et al. 2004). The role of MAPK signalling in inducing plant systemic resistance during *Trichoderma*-plant interaction was recently examined using *T. virens* Δ*tmkA* mutants. When *Trichoderma* spores were germinated in proximity to cucumber roots, Δ*tmkA* mutants were able to colonize the plant roots as effectively as the wild type strain but they failed to induce full systemic resistance against the bacterial leaf pathogen *Pseudomonas syringae* pv*. lacrymans* suggesting that *T. virens* needs MAPK signalling in order to induce full systemic resistance in the plant (Viterbo et al. 2005).

*T. harzianum* ThHog1, which is highly similar to Hog1p controlling the osmotic stress response in *S. cerevisiae* (Brewster et al. 1993), was recently characterized (Delgado-Jarana et al. 2006). ThHog1 was shown to be phosphorylated under different stress conditions such as hyper-osmotic or oxidative stress and the protein was demonstrated to be localized in nuclei under these stress conditions. The main role of ThHog1 was proposed to be in the hyper-osmotic stress response since a *hog1*-silenced mutant was highly sensitive to osmotic stress and showed intermediate levels of resistance against oxidative stress (Delgado-Jarana et al. 2006). A *T. harzianum* mutant strain carrying a *hog1* ^F315S^ allele was highly resistant to the calcineurin inhibitor cyclosporine A, suggesting links between the two pathways. In plate confrontation assays the two mutant strains had strongly reduced antagonistic activity against the plant pathogens *Phoma betae* and *Colletotrichum acutatum* but no changes in the mycoparasitic ability against *B. cinerea, R. solani* and *S. sclerotiorum*. It was proposed that ThHog1 could be involved in neutralizing stress agents, such as reactive oxygen species, produced by the parasitized fungi during the mycoparasitic action (Delgado-Jarana et al. 2006).

## cAMP Signalling During *Trichoderma* Biocontrol

In fungi, cAMP signalling is involved in a variety of processes including the control of differentiation, sexual development, virulence, monitoring of the nutrititional status, and stress. The cAMP pathway also influences transcription and cell cycle progression (Kronstad et al. 1998). cAMP levels are regulated by a membrane-associated adenylate cyclase for synthesis and a cAMP-specific phosphodiesterase for degradation. Activity of adenylate cyclase, which synthesizes the intracellular messenger cAMP, has been shown to be regulated by α subunits of heterotrimeric G-proteins in most fungi. Most of the effects of cAMP in eukaryotes occur via the stimulation of cAMP-dependent protein kinases (PKA) which consist of two regulatory and two catalytic subunits (Dickman and Yarden, 1999).

In plant pathogenic fungi, processes like growth, morphogenesis, and virulence are known to involve functional PKA (e.g. Xu and Hamer, 1996; Dürrenberger et al. 1998).

cAMP was reported to act as a positive effector of endoglucanase induction by enhancing the efficacy of the induction process in *T. reesei,* a species which was recently shown to be able to antagonize and overgrow *Pythium ultimum* and to provide protection of zucchini plants against *P. ultimum* blight in planta (Sestak and Farkas, 1993; Seidl et al. 2006). In *T. harzianum,* application of exogenous cAMP increased coiling around nylon fibres in the biomimetic system (Omero et al. 1999) and substances which increase the intracellular levels of cAMP (e.g. dinitrophenol, caffeine, aluminium tetra fluoride) repressed the synthesis of N-acetyl-β-D- glucosaminidase (Silva et al. 2004).

The role of cAMP signalling during conidiation was investigated in *T. viride* and *T. atroviride* (Nemcovic and Farkas, 1998; Casas-Flores et al. 2006). The production of conidia is the main mechanism for *Trichoderma* to survive and disperse in the environment and conidiation is induced by environmental factors such as blue light and nutrient stresses in these mycoparasites. In *T. viride*, the photoinduction of conidiation is accompanied by a rapid temporal rise in the intracellular level of cAMP (Gresik et al. 1988), and exogenous cAMP stimulated the formation of conidia in both illuminated colonies and in colonies that were kept in the dark (Nemcovic and Farkas, 1998). Recently, a gene (*pkr-1*) encoding the regulatory subunit of protein kinase A (PKA) from *T. atroviride* was cloned and characterized and the authors demonstrated that PKA plays an important role in the regulation of light responses in this fungus (Casas-Flores et al. 2006). Mukherjee et al. (2007) recently reported on the cloning of an adenylate cyclase-encoding gene (*tac1*) of *T. virens*, whose deletion lowered the intracellular cAMP levels to below the detection limit. Δ*tac1* mutants showed heavily reduced growth rates on agar, did not sporulate in darkness, were unable to overgrow host fungi like *S. rolfsii*, *R. solani*, and *Pythium* sp., and exhibited reduced secondary metabolite production.

## Conclusions

Mycoparasitism is a fungus-fungus interaction comprising host-pathogen cross-talk. Until now, only little information has been available on the role of conserved eukaryotic signalling pathways during this interaction.

Isolation and characterization of selected components involved in different signal transduction pathways of mycoparasitic and biocontrol-active *Trichoderma* strains recently started and revealed high sequence conservation to homologous proteins from other fungi.

Signalling pathways involving G-protein α subunits as well as mitogen-activated protein kinases have been repeatedly shown to be important for virulence in both plant pathogens as well as mycoparasites. Therefore, G protein-coupled receptors are promising targets as they are putatively involved in host recognition and may be responsible for triggering responses resulting in e.g. host attack.

**Figure 1:**
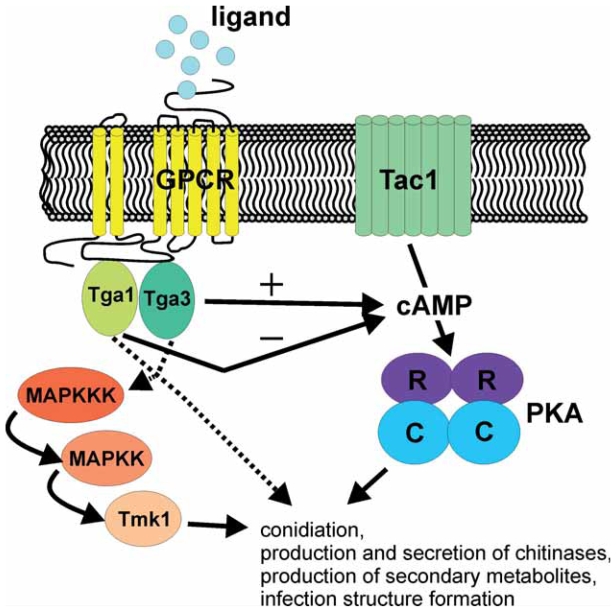
Schematic representation of selected components of mycoparasitism- related signalling pathways of *T. atroviride*. Tga1, Tga3: subgroup I and III G-protein α subunits; GPCR: G protein-coupled receptor; Tac1: adenylate cyclase; PKA: protein kinase A regulatory (R) and catalytic (C) subunits; Tmk1: MAP kinase

Research on G-protein signalling in *Trichoderma* spp. revealed that they have members of each fungal Gα subgroup. In *T. atroviride*, both the subgroup I and III Gα subunits Tga1 and Tga3 directly affect mycoparasitism-related processes by having overlapping roles in the regulation of mycoparasitism-related genes (Fig. 1). For both Tga1 and Tga3, an interaction with the cAMP pathway was found, as the Δ*tga1* mutant exhibited an elevated internal cAMP level, whereas Δ*tga3* mutants had reduced internal cAMP levels compared to the wild type control. Although not all processes contributing to mycoparasitism could be restored by exogenous cAMP in the *T. atroviride* G protein mutants, it was shown that Tga1 affected antifungal metabolite production by signalling via the cAMP pathway, whereas Tga3 is involved in infection structure formation in a cAMP-dependent manner. Furthermore, PKA was shown to play an important role in the regulation of light responses in *T. atroviride,* and adenylate cyclase was reported to be involved in growth, germination and mycoparasitism in *T. virens*.

In addition to Gα proteins and the cAMP pathway, the *T. atroviride* MAPK Tmk1 (Fig. 1) and the *T. virens* MAPK TmkA/Tvk1 also directly affect transcription of mycoparasitism-related genes, suggesting that Tga1/TgaA and Tga3 could also be connected with the Tmk1/TmkA-containing MAP kinase cascade in regulating *Trichoderma* mycoparasitism-associated gene transcription.
